# Impact of Pre-Elite Anterior Cruciate Ligament (ACL) Reconstruction on Draft Outcomes and Reinjury Risk in Elite Australian Football Players: A Retrospective Cohort Study

**DOI:** 10.3390/sports13090308

**Published:** 2025-09-05

**Authors:** James P. Veale, Anik Shawdon, Peter D’Alessandro, Jay R. Ebert, Peter K. Edwards

**Affiliations:** 1 Australian Football League, Melbourne, VIC 3001, Australia; 2 Orthopaedic Research Foundation of Western Australia, Perth, WA 6010, Australia; 3 Fiona Stanley and Fremantle Hospitals Group, South Metropolitan Health Service, Perth, WA 6150, Australia; 4 School of Surgery, University of Western Australia, Perth, WA 6009, Australia; 5 School of Human Sciences (Exercise and Sport Science), University of Western Australia, Perth, WA 6009, Australia; 6 School of Allied Health, Curtin University, Perth, WA 6102, Australia

**Keywords:** anterior cruciate ligament, reinjury, family history, draft, Australian Football, ACL tear

## Abstract

A history of anterior cruciate ligament reconstruction (ACLR) during the Australian Football (AF) talent pathway may impact draft prospects and increase the risk of a second injury during a professional career. This study investigated whether a history of ACLR before the Australian Football League (AFL) Draft Combine influenced draft outcomes and second ACL injury rates during an AFL career. A retrospective cohort study was undertaken, whereby AFL talent pathway medical evaluation data from 1071 male players who attended the AFL National Draft Combine between 2011 and 2022 was analyzed. Draft outcomes and secondary ACL injuries were compared between players with and without a pre-draft ACLR using chi-square and Fisher’s exact tests. Twenty-one players (2.0%) had sustained a pre-draft ACLR. All of these players were drafted into the AFL. Players with a prior ACLR were more likely to be drafted in the first two rounds (odds ratio [OR] 2.64; *p* = 0.039). They were also over eight times more likely to sustain another ACL injury during their careers (28.6% vs. 4.5%; OR 8.54; *p* < 0.001). This study showed that a pre-draft ACLR did not reduce the likelihood of being drafted but was associated with a higher risk of a secondary ACL injury during an AFL career. These findings are relevant to AFL recruiters, medical staff, and high-performance staff, and support the consideration of education and injury prevention programs for pre-elite players with prior ACLR.

## 1. Introduction

Australian football (AF) is a fast-paced team sport played from junior to elite-level competitions of the Australian Football League (AFL) and AFL Women’s (AFLW). The multidirectional demands of the sport involve frequent landing, cutting, and pivoting movements, which increase the risk of anterior cruciate ligament (ACL) injury [1,2,3,4]. In the AFL, the incidence rate of ACL injuries ranges from 0.38 to 1 event per team per season [5,6,7] and consistently ranks among the leading causes of missed games in a season [1]. The AFL talent pathway identifies and develops pre-elite players [8] who progress through representative underage programs and may be invited to the AFL National Draft Combine [9,10]. This multi-day event involves physical testing, medical evaluations, psychological assessments and club interviews, which club recruiters use to help inform draft decisions [11]. Importantly, this development phase coincides with peak ACL injury risk, particularly in males 15–19 years who have one of the highest ACL injury rates nationally (241 per 100,000) [12]. An ACL injury, and subsequent ACLR, before a player has been drafted may impact their draft prospects and their future AFL career.

In other professional sports such as American Football, pre-draft ACL injuries have been associated with draft selection in later rounds, reduced early-career participation in matches, and higher rates of subsequent ACL injury [13,14]. In elite AF, while return to sport rates after ACL reconstruction (ACLR) are good [3], the risk of graft rupture or a contralateral ACL injury is also high, particularly in young players. Previous studies have reported reinjury rates as high as 44% in males younger than 18 years [15,16,17]. This is particularly relevant to pre-elite AF players most of whom are adolescents or emerging adults and may be entering the elite system during the period of highest reinjury risk. Beyond individual injury history, family history of ACL injury has also been identified as an independent risk factor, with meta-analyses estimating a 2.5-fold higher risk of primary and subsequent injury [18], and recent data in elite AF players showing more than a threefold increase [19]. It remains unclear, however, to what extent family history of ACL injury is incorporated into talent identification or injury monitoring systems within AF.

Physical test performance at the AFL Combine has been well-studied in relation to draft outcomes and career success [11,20,21,22]. Whether AFL clubs draft players with a history of ACLR, and the extent to which these players face an increased risk of future ACL injury, has not been established. No study has specifically investigated the influence of ACLR sustained during the AFL talent pathway on draft selection or subsequent ACL injury risk. In addition, family history of ACL injury has been linked to primary ACL injury risk in elite players, but its influence during the transition from pre-elite to elite AFL competition is not well understood. The aim of this study was to determine whether a pre-draft ACLR influenced draft outcomes and reinjury risk in male AFL players. Specifically, the study examined (1) whether a history of ACLR during development and pre-elite years influenced draft success; (2) whether a history of ACLR during development and pre-elite years increased the risk of sustaining a secondary ACL injury during a professional AFL career; and (3) whether family history of ACL injury was associated with either pre-draft ACLR or an ACL injury during a professional AFL career.

## 2. Materials and Methods

A retrospective review was conducted for all players who attended the AFL National Draft Combine between 2011 and 2022. Each player completed a standardized medical assessment, which recorded any personal history of ACL injury and family history of ACL injury in first-degree relative. All players that were eligible to participate in the AFL National Draft Combine during this period were included. Written information was provided to all participants at the time of completing the standardized medical evaluation form and provided (along with a parent/guardian in the event a player was under the age of 18 years when completing the questionnaire) written informed consent for their data to be used for the purposes of research. Approval to use the data for research purposes was granted by the AFL and the AFL Research Board, with ethics approval obtained from the Human Research Ethics Committee of the University of Western Australia, Perth, Australia (2024/ET000462).

### 2.1. Data Collection and Analysis

Pre-draft information on ACL injury and ACLR obtained from the AFL National Draft Combine Medical Evaluation Forms of 1071 male AF players (mean age 18.6 ± 0.9 years) between 2011 and 2022 (inclusive). Relevant information from these forms included player responses to questions relating to having a history of ACLR, as well as whether anyone from their immediate family had a history of ACL injury. Draft outcomes, including which draft a player was taken in (National Draft, Pre-season Draft, or Rookie Draft), what draft round they were taken in, and what overall selection number they were drafted, was determined by cross-referencing the study group with the AFL Draft Order records between 2011 and 2022, obtained from AFL Historical Statistics and stored in Microsoft Excel format. Players were classified as either drafted or not drafted into the AFL. ACL injuries that were sustained during an AFL career were identified through the league’s official injury surveillance system, which underpins the AFL’s annual injury reports [5,23]. This data was reviewed for the period 2012 to 2023 using information published each year in the AFL Season Record.

### 2.2. Statistical Analysis

All analyses were performed on a complete retrospective dataset, with no missing value for the included variables. Descriptive statistics were performed and presented as means and standard deviations, for continuous variables, and frequencies and percentages for categorical variables. Pre-draft ACL injuries, a family history of ACL injury, draft selection and an ACL injury sustained during a player’s career was treated categorically (yes/no). Draft round was recorded as an ordinal variable (1st, 2nd, etc.) based on official AFL Draft records. Comparisons between variables were assessed with the chi-square or Fisher’s exact test for categorical variables, with odds ratios (ORs) and 95% confidence intervals (CIs) reported. The strength of associations was also reported using Cramer’s phi (V), with values categorized as >0.25 (very strong), 0.15–0.25 (strong), 0.10–0.15 (moderate), 0.05–0.10 (weak), and 0–0.05 (no/very weak association). Statistical significance for all analyses was set at *p* < 0.05. All statistical analyses were conducted using SPSS (version 30, IBM Corp., Armonk, NY, USA).

## 3. Results

Out of the 1071 AF players included in this retrospective analysis, 793 players (74.0%) were successfully drafted into the AFL. Of this, 61.1% (n = 654) were selected in the National Draft, while 13.0% (n = 139) were taken in secondary drafts (the Rookie Draft and the Pre-season Draft). Two per cent of players (n = 21) had undergone an ACLR as a pre-elite athlete, and all were subsequently drafted. Of the 21 players who sustained an ACL injury as a pre-elite athlete, 15 (71.4%) were selected in the first or second round of the AFL draft, while 6 (28.6%) were selected in later rounds. Players undergoing ACLR as a pre-elite athlete were significantly more likely to be selected in earlier rounds (1 or 2) of the AFL draft compared to those without such an injury history (OR = 2.64; 95% CI, 1.02–6.85, *p* = 0.039), although this association was weak (Cramer’s phi = 0.063).

A total of 59 ACL injuries were reported in 53 AFL players after being drafted, from 2011 to 2022 (Table 1). All 21 players with a pre-draft ACL injury had undergone ACLR, as confirmed in AFL Draft Combine medical evaluations. For ACL injuries sustained post-draft, due to the de-identified nature of the dataset, information on whether these cases were surgically managed or not, was not known. Players with a pre-draft ACLR had 8.54 times greater odds (95% CI [3.17, 22.99]) of experiencing another ACL injury after being drafted into the elite-level AFL compared to those without a prior ACLR (28.6% vs. 4.5%; χ^2^ = 25.4, *p* < 0.001) (Table 1).

Table 2 shows the frequency of players with a family history of ACL injury and the occurrence of an ACL injury as a pre-elite athlete. No significant association was observed between players with a family history of ACL injury and sustaining an ACL injury as a pre-elite athlete (4.2% vs. 1.7%; *p* = 0.074). Additionally, no significant association was observed in players between having a family history of ACL injury and experiencing an ACL injury during an elite AFL career (20.8% vs. 11.8%; *p* = 0.079) (Table 3).

## 4. Discussion

The aim of this study was to determine whether having a history of ACLR sustained before the AFL Draft Combine was associated with draft outcomes and the risk of secondary ACL injury during a professional AFL career. The main findings were that players with a prior ACLR were still commonly drafted, including in early rounds, but had a higher likelihood of sustaining another ACL injury during their AFL career. Family history of ACL injury was not associated with an increased risk of injury either before or after being drafted. Given the high burden of ACL injuries in the AFL, these findings provide insight into the role of both individual and family history in the talent pathway and their influence on career outcomes [1,5,6].

The results of this study are consistent with previous studies showing that ACL injuries sustained before 18 years of age carry a relatively high risk of recurrence [15,17,24]. Out of the players drafted, approximately 7% ended up experiencing an ACL injury during their AFL career, and of those, approximately one-third had undergone ACLR prior to being drafted. This represents approximately an 8.5 times greater likelihood of an ACL injury in those with a prior ACLR, compared to those without. Despite this increased risk, having a history of ACLR did not reduce the chance of being drafted. In fact, players with a prior ACL injury were more likely to be drafted within the first two rounds of the National Draft. This contrasts with evidence from the NFL, where athletes with a history of ACLR were drafted later than uninjured peers [13]. One explanation may be that AFL recruiters place less weight on injury history and greater weight on factors such as game performance, perceived potential, team balance, and list management needs [25]. Furthermore, the physical performance profiles of players when tested at the AFL Draft Combine have previously shown limited value in predicting injury risk, in part because all players at this stage share similar physical traits and athletic profiles [11]. Previous studies have also suggested that match performance, more than physical testing results, distinguishes players who are drafted from those who are not [26]. Therefore, it seems that club recruiters place more weight on player traits such as ability or skill, rather than physical capacity or injury history. On the other hand, players with lower ability who sustain an ACL injury may slip from draft consideration through lost development time, whereas stronger players are more likely to recover, return, and remain visible to recruiters.

Advances in surgery and rehabilitation have improved return-to-play rates and career longevity after ACL injury [27,28,29]. Even so, identifying players at higher risk earlier in their development remains important for planning and applying prevention strategies or injury “resilience” programs. In this study, players who sustained an ACL injury and underwent ACLR during the talent pathway were more than eight times more likely to suffer a subsequent ACL injury during their AFL career. Evidence supports the use of exercise-based, and particularly strength-based interventions to reduce lower limb injury risk [30,31,32]. Injury prevention programs have been effective in community-based AFL [33], and in junior athletes [34], but the effect of ACL-specific programs in the AFL talent pathway remains unclear. These findings support the consideration of implementing injury prevention programs for young players within an AFL talent pathway, especially those with a history of an ACLR, with a focus on modifiable risk factors to reduce the risk of a secondary ACL injury.

Our findings differ from previous studies that show stronger associations between family history and ACL injury risk. [18,35]. A recent meta-analysis has shown a 2.5-fold increased risk of primary ACL injury, and a 2.4-fold increased risk of reinjury in those with a family history [18]. In addition, it has been shown that male elite-level AF players with a family history of ACL injury were over three times more likely to sustain an ACL injury than those without [19]. Family history is a risk factor that can change from not being present to being present, therefore the lack of significant association in our study may be because family history was recorded once during medical screening prior to being drafted at the AFL Draft Combine. This may underestimate the true family history of a player, as injuries in relatives can occur later through a player’s AFL career. While the results of the current study did not find a significant association, regular screening for a family history of injury may be one way to identify athletes who are at a higher risk of ACL injury, as has been previously recommended [19].

The results from this study might be relevant to AFL recruiters and high-performance and medical staff of AFL clubs. For example, players with a history of ACLR are almost always drafted, including in the early rounds, but they also carry a higher risk of reinjury. If recruiters are considering players with certain qualities that make them attractive irrespective of their injury history, injury prevention programs and player education might be a consideration for those with a history of ACLR, to reduce the risk of a secondary ACL injury during their career.

This study has several limitations. First, it was retrospective and limited to pre-elite AF athletes who attended the Draft Combine. This design was intentional given the focus on the talent pathway, but it may be viewed as a form of sampling bias. Second, the small number of players with a pre-draft ACLR (n = 21) introduces uncertainty into the effect estimates, as reflected in the wide confidence intervals. Because this was a retrospective study, no sample size estimation or power analysis was performed, which might be considered a limitation. Third, information on family history was self-reported at the time of the medical evaluation using a structured questionnaire often completed with parental assistance. This likely improved accuracy compared with recall later in a professional career, but the validity of self-reported family history cannot be confirmed. Fourth, only male AF players were included, reflecting the longer-established history of the men’s competition and the Draft Combine. With the recent growth of the AFLW, future research should investigate the influence of pre-draft ACL injury and family history in female players. Fifth, the dataset did not provide details on surgical management of post-draft ACL injuries, or on intraoperative features of pre-draft ACLRs such as graft type, meniscal injury and repair, or the use of lateral extra-articular tenodesis. Data on player position, match performance, and physical qualities such as strength, speed, and agility were not available in this study. These factors are likely to influence both draft outcomes and injury risk. Future work that includes these measures would allow more accurate modeling of draft selection and reinjury risk. Further research into mechanisms of injury and the role of prevention programs is needed to reduce reinjury rates and support longer careers in AFL players. Finally, as this study focused on association and not on causation of ACL injuries, future research investigating the injury mechanisms and the role of targeted injury preventative programs would provide significant benefits to reducing the injury risk and increase the career longevity of future AFL players.

## 5. Conclusions

Having a history of ACLR prior to being drafted did not reduce the likelihood of players being drafted into the AFL but was associated with a higher risk of a secondary ACL injury during their professional AFL career. Larger studies that include other variables, such as physical attributes and career performance metrics, are needed to confirm these findings, assess the influence of other potential risk factors, and determine the subsequent effect on career longevity and match performance at the elite level. Injury history should remain an important part of draft screening, and targeted prevention strategies for pre-elite players with prior ACL injury should be considered.

## Figures and Tables

**Table 1:** Frequency of an ACL reconstruction (ACLR) prior to draft age and future AFL Career ACL injury (n = 1071).

AFL Career ACL Injury	ACLR Pre-Draft (n = 21)	No ACL Injury Pre-Draft (n = 1050)	Total
Yes	6 (28.6%)	47 (4.5%)	53 (4.9%)
No	15 (71.4%)	1003 (95.5%)	1018 (95.1%)
Total	21 (100%)	1050 (100%)	1071 (100%)
Odds Ratio (95% CI)	8.54 (3.17–22.99)		
*p* Value	*p* < 0.001		

**Table 2:** Association between family history of ACL injury and likelihood of pre-draft ACL injury among AFL draft combine participants (n = 1071).

Family History of ACL Injury	ACL Injury Pre-Draft (n = 21)	No ACL Injury Pre-Draft (n = 1050)	Total
Yes	5 (4.2%)	114 (95.8%)	119 (4.9%)
No	16 (1.7%)	936 (98.3%)	952 (95.1%)
Total	21 (100%)	1050 (100%)	1071 (100%)
Odds Ratio (95% CI)	2.57 (0.92–7.14)		
*p* Value	0.074		

**Table 3:** Association between family history of ACL injury and incidence of ACL injury during an elite-level AFL career (n = 793).

Family History of ACL Injury	AFL Career ACL Injury (n = 53)	No AFL Career ACL Injury (n = 1018)	Total
Yes	11 (20.8%)	87 (11.8%)	98 (12.4%)
No	42 (79.2%)	653 (88.2%)	695 (87.6%)
Total	53 (100%)	740 (100%)	793 (100%)
Odds Ratio (95% CI)	1.97 (0.98–3.96)		
*p* Value	0.079		

## Data Availability

The original contributions presented in the study are included in the article, further inquiries can be directed to the corresponding author.
